# Emotion-based design research of rural street spaces using eye-tracking technology: A case study of Huixingtou Village in Handan City

**DOI:** 10.1371/journal.pone.0326049

**Published:** 2025-06-25

**Authors:** Ruili Wang, Fan Yang, Qingqin Wang

**Affiliations:** 1 School of Architecture and Art, Hebei University of Engineering, Handan, China; 2 The State Key Laboratory of Building Safety and Built Environment, China Academy of Building Research, Beijing, China; Shenyang Jianzhu University, CHINA

## Abstract

Rural street spaces serve as primary venues for communal activities, yet emotion-based design in these spaces remains underexplored. This study delineates three typical scales of rural street spaces in China northern plains region, utilizing eye-tracking technology, investigates the constituent elements and materials of various optimized design schemes, analyzing people’s emotional perceptions of different elements and materials. The results indicate that: (1) narrower streets evoke a greater sense of security among individuals; (2) an increased variety and quantity of paving materials, landscape flower beds, seating areas, and public facilities heighten people’s visual interest, enhancing the spatial publicness and safety; (3) higher coverage of green landscape relaxes visual perceptions, leading individuals to linger and dwell in the space. Consequently, through judicious design of scale, constituent elements, and materials, rural street spaces can be effectively imbued with emotional expressions, thereby elevating the spatial quality of rural street spaces to meet people’s emotional needs.

## 1. Introduction

With the accelerated pace of urbanization, the pursuit of a better quality of life and higher-quality spaces has become a prominent theme in urban and rural development [1]. Rural areas constitute an integral part of societal construction, and the optimization and improvement of rural street spaces directly influence people’s quality of life and sense of well-being [2]. Rural street spaces, as essential nodes of social interaction and daily activity, significantly impact community cohesion and individual well-being. However, research on integrating emotional design principles into rural streetscapes remains limited. Emotional design, as the term suggests, is an approach that aims to evoke positive emotions in users through design, creating environments that are not only functionally efficient but also capable of eliciting feelings of pleasure, security, or comfort. In his book *Emotional Design: Why We Love (or Hate) Everyday Things*, Norman (2004) proposed three levels of emotional design: visceral (an immediate sensory response, such as visual appeal), behavioral (performance-based usability, such as ease of use), and reflective (deep cognitive processing related to meaning, such as connections to personal or community identity) [3]. In the context of rural street spaces, emotional design implies that streets should not only fulfill transportation and social interaction functions but also incorporate elements such as materials, colors, and greenery to evoke positive emotions, enhancing users’ sense of security, comfort, and community belonging. In urban design, studies have shown that certain design qualities influence emotional responses. Ewing and Handy (2009) identified that imageability, enclosure, and human scale enhance walkability, which is closely associated with positive emotional experiences [4]. In landscape design, natural elements are considered crucial for emotional restoration. Kaplan (1989) proposed that exposure to natural environments provides restorative experiences, reduces stress, and enhances emotional well-being [5]. White et al. (2013) further emphasized that natural landscapes, such as water features, can amplify positive emotional responses, which may also apply to the greening strategies of rural street spaces [6]. However, rural street spaces differ significantly from urban streets and landscape areas. Rural streets are typically narrower, feature more greenery, and foster stronger community connections, potentially requiring adjustments to emotional design principles. For instance, while urban safety perception may rely more on visibility and lighting, rural safety perception might be more closely linked to familiarity and community presence. Additionally, rural streets often carry deeper historical and cultural significance, making them more likely to evoke emotional resonance at the reflective level of emotional design.

This study aims to bridge this gap by investigating the interplay between spatial design elements and emotional experiences within rural street environments. Therefore, explore the relationship between the design elements of rural street spaces and emotional perceptions of spatial experiences [7], with a particular focus on people’s pursuit of safety, comfort, convenience, and social interactions in rural street environments. Adhering to a people-oriented approach, the study conducts emotion-based evaluations of rural street space designs from the perspective of individual spatial perception of space. Recent studies have highlighted the importance of integrating cultural, ecological, and emotional dimensions in spatial design to create meaningful and sustainable environments [8]. In the context of rural street spaces, these dimensions are particularly relevant as they shape the unique character and user experience of these areas. This research aims to provide more scientific and refined guidance for the design of rural street spaces and the planning of social spaces [9].

Street spaces, especially those with high functional complexity like rural street spaces, play a crucial role in facilitating interpersonal interactions and building social relationships. As vital open spaces in rural areas, rural streets are characterized by high usage rates, a significant proportion of community land occupancy, and strong permeability, serving as essential components of flexible interaction spaces. Wang et al in their recent study found that, in the North China Plain, rural streets are essential for transportation and social interaction, with main roads used for vehicles and alleys for walking. These streets occupy a significant proportion of community land, and their design often ensures strong permeability, allowing for easy movement within the village [10]. Kevin Lynch, in The Image of the City, mentions that both urban and rural streets serve as carriers of collective memory and social space [11]. Sidewalks on both sides of the street, roads in front of residential houses, and roads on both sides of residences are pivotal spatial carriers that support neighborly interactions [8]. The spatial form, interfaces, and nodes of street spaces play a significant role in promoting social activities [12]. Improvements in street space facilities, such as resting amenities, plant greening, traffic safety installations, and ground paving, can effectively enhance street usage and interaction behaviors [13].

The constituent elements of street spaces serve as pivotal material carriers that most effectively promote social activities, constituting essential material elements for rejuvenating and invigorating social interactions and enhancing street space vitality [14]. The quality of street spaces significantly influences their vitality, with evaluation indicators for spatial quality encompassing facility density, accessibility, sky openness, and enclosure degree among other metrics [15]. Different constituent elements of street spaces also affect the distribution characteristics of street space vitality [16]. Currently, researchers employ artificial intelligence technologies to perceive urban street spaces and establish facade prediction models to assist in generating localized street design schemes [17]. Simultaneously, design elements such as functional zoning, pedestrian width, and interface openness in street spaces significantly influence people’s travel behaviors [18].

The constituent elements of street spaces influence individuals’ perceptions within these spaces. Research indicates that factors such as the functionality, interface, green view ratio, and physical environment of street spaces impact individuals’ sense of security within these environments [19]. Physical space, ground-level interfaces, greening facilities, and macro forms of streets positively influence perceptions and evaluations of spatial aesthetics [11]. The virtual-real relationship and rhythm of street interfaces also play a significant role in pedestrians’ psychological cognition and spatial perception [20].

Emotion-oriented design has become a significant focus in contemporary spatial and architectural studies, as spaces are increasingly recognized not only as functional environments but also as stimuli for emotional responses. This trend is particularly relevant in rural street spaces, where the integration of cultural [21], ecological [22], and emotional [23] dimensions shapes users’ experiences [24]. The following review synthesizes recent interdisciplinary research on emotional perception in urban and rural environments, with an emphasis on methodologies and findings that inform the design of rural street spaces.

Urban studies provide foundational insights into how built environments affect emotions. Xiang et al. (2021) demonstrated that visual exposure in dense urban areas, combined with machine learning, can model pedestrian emotions. Their real-time tracking of physiological and psychological responses highlights the significance of visual elements in shaping emotional experiences [25]. Similarly, Zhu and Xu (2021) analyzed sentiment in urban green spaces during COVID-19, finding that emotional demand for open spaces increased in times of social stress [26]. These urban-focused studies underline the importance of designing visually and emotionally stimulating environments, concepts that can be extrapolated to rural contexts.

The role of color and landscape features is crucial in emotional design. Zhang et al. (2024) explored how neighborhood color impacts children’s emotional mentalization, employing multi-source data to evaluate these effects. Their findings highlight the potential of color as a design tool in shaping emotional well-being [27]. Similarly, Sun et al. (2024) assessed emotional perceptions of waterfront greenways, emphasizing the relationship between landscape features and user satisfaction. These findings underscore the importance of integrating natural and cultural elements into rural street spaces to evoke positive emotions [28].

Microscale methods provide nuanced insights into emotional dynamics within specific spatial contexts. Fu and Chan (2019) proposed a microscale measurement method for Beijing hutongs, combining spatial design with emotional evaluations to develop a deeper understanding of users’ experiences [29]. This approach can inform the design of rural street spaces.

The role of nature in emotion regulation is increasingly acknowledged, where intimate interactions with the environment are similarly critical. Vitale and Bonaiuto (2024) conducted a rapid review demonstrating the impact of natural environments on emotional regulation, emphasizing biophilic design principles that integrate natural elements [23]. Similarly, Van Stokkum (2022) investigated how sociocultural dimensions, such as cycling advocacy, influence emotional experiences within transportation systems [30]. Therefore, when implementing emotional design in rural street spaces, optimizing natural environments and cultural elements can enhance people’s emotional experiences. As Fu et al. (2022) conducted a comprehensive study on the impact of visual and soundscape design in rural street spaces on the therapeutic effects of rural community streets. The results indicate that street space typology influences healing potential, with artificial-natural enclosed streets and natural semi-enclosed streets exhibiting the highest therapeutic effects [31]. Zhang (2024) explored the intersection of architecture and emotions through sensory dynamics and methodological innovations, advocating for a multidisciplinary approach to studying spatial environments [27]. These principles are vital for advancing the design of rural street spaces, where cultural heritage and sensory diversity play key roles in shaping emotional experiences.

Prior studies have highlighted the influence of green landscapes, spatial configurations, and public amenities on user perceptions of safety, aesthetics, and sociability. Building on these insights, this research adopts an interdisciplinary approach, combining eye-tracking technology with subjective assessments to analyze emotional responses to different street configurations and design elements. Eye tracking technology is employed in this study to capture real-time, objective data on individuals’ visual attention, which is crucial for understanding their emotional responses to different rural street space configurations. Unlike traditional subjective self-reports, eye tracking provides precise measurements [32] of gaze patterns, fixation durations, and saccade movements [8]. This allows us to analyze how different street elements, such as greenery, paving materials, and public facilities, engage people’s attention and influence their emotional experiences. By combining eye tracking with subjective assessments, we can gain a more comprehensive understanding of the relationship between the design elements of rural street spaces and people’s emotional perceptions.

This study fills a research gap in the emotional design of rural streets by utilizing eye-tracking technology to measure visual attention, infer emotional interest, and explore how design elements influence user experience. This focus differs from that of urban or landscape studies, where urban research primarily emphasizes traffic flow and commercial facilities, while landscape studies are more concerned with natural restoration. In contrast, rural street design must balance community interaction, natural elements, and cultural heritage. Wang et al. (2020) highlighted the significance of community engagement in enhancing residents’ well-being [33]. Furthermore, Panzera pointed out that cultural heritage has been shown to be strongly associated with a broader sense of community belonging [34].

This study aims to conduct an in-depth analysis of the visual attractiveness, pleasure, and emotional evaluations of subjective perceptions of rural street spaces influenced by different constituent elements through visual perception experiments and subjective questionnaire surveys. By examining elements such as material colors, green landscapes, and public facilities in rural street spaces, the study explores their mechanisms in meeting people’s emotional needs [35]. Understanding the relationship between the constituent elements of rural street spaces and people’s emotional requirements can provide scientific foundations and practical guidance for emotion-based design of rural street spaces in the future. Focusing on Huixingtou Village as a case study, this work seeks to identify practical strategies for designing rural street spaces that prioritize emotional well-being and foster vibrant, sustainable communities.

## 2. Research methodology

How do different constituent elements of street spaces influence emotional perceptions and interaction behaviors within those spaces? How can the design of street spaces promote the occurrence of interaction behaviors and enhance people’s emotional identification within these spaces? To explore these questions, we employed a combined approach of objective eye-tracking experiments and subjective emotional questionnaires to analyze people’s perceptions of emotion-based street space design, their preferences for compositional elements, and the underlying cognitive processes. Building upon eye-tracking data of participants observing and recognizing street space environments, the experiment incorporates questionnaire surveys.

The objective eye-tracking experiment utilized eye-tracking equipment to record and analyze participants’ physiological eye movement data while observing spatial design images. This approach provides an objective and scientific assessment of how different rural street space design elements influence emotional perception through physiological responses.

The subjective questionnaire employed an emotional survey to assess participants’ emotional satisfaction after viewing images of various rural street space designs. An emotional scale was used to analyze individuals’ subjective emotional perceptions of different design elements.

The experiment aims to investigate the impact of different design elements of rural street space on the overall perception and public activities within rural street space. Through an integrated analysis of subjective and objective methods, we comprehensively examine the impact of street space design on people’s emotional perceptions.

### 2.1. Selection and design of rural street spaces

#### 2.1.1. Selection of rural street spaces.

Rural street space, as a frequently utilized venue for daily public activities in rural areas, exerts different influences on the types of public activities based on various street scales. We selected Huixingtou Village in Kangzhuang Township, Fuxing District, Handan City, Hebei Province, as the case study for this research. The village features a typical rural street layout of the North China Plain and includes three primary scales and types of rural streets found in northern China, making it highly representative. Additionally, its location at the urban-rural interface results in a relatively large permanent population, leading to more diverse street activities and stronger social interaction demands. Through preliminary research on the street space of Huixingtou Village, we categorize the village’s street space into three types: main roads, side roads, and front-of-house roads, based on width, segregation of pedestrian and vehicular traffic, and flower bed configurations, as depicted in Fig 2. Main roads have a width of ≥6 meters, segregated pedestrian and vehicular traffic, and are flanked by sidewalks and flower beds on both sides. Side roads are 4–5 meters wide, allowing mixed pedestrian and vehicular traffic, with no flower beds or sidewalks on either side. Front-of-house roads are 5–6 meters wide, allowing mixed pedestrian and vehicular traffic, with flower beds on one side and no sidewalks.

**Fig 1:**
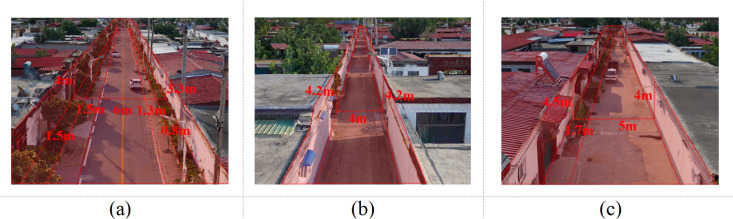
Types of rural street spaces. (a) Main Road (Width ≥6 meters, with sidewalks and flower beds on both sides); (b) Side Road (Width 4-5 meters, mixed pedestrian and vehicular traffic, without flower beds); (c) Front-of-house Road (Width 5-6 meters, mixed pedestrian and vehicular traffic, with flower beds on one side).

Through observation of residents’ activities in these three types of street space throughout the day, it was found that although there are sidewalks and flower bed landscape facilities on the main road, people tend to engage less in social activities and more in front-of-house road spaces where they stop and socialize. While the side road space is narrower, it experiences higher pedestrian traffic. Residents tend to engage in social activities, such as chatting and conversing, at the intersections of side roads and front-of-house roads. In order to comprehensively investigate residents’ willingness to engage in different interaction activities and their spatial preferences in the main road, side road, and front-of-house road spaces, we conducted separate experiments for each of these three types of street space.

#### 2.1.2. Stimuli.

**Fig 2:**
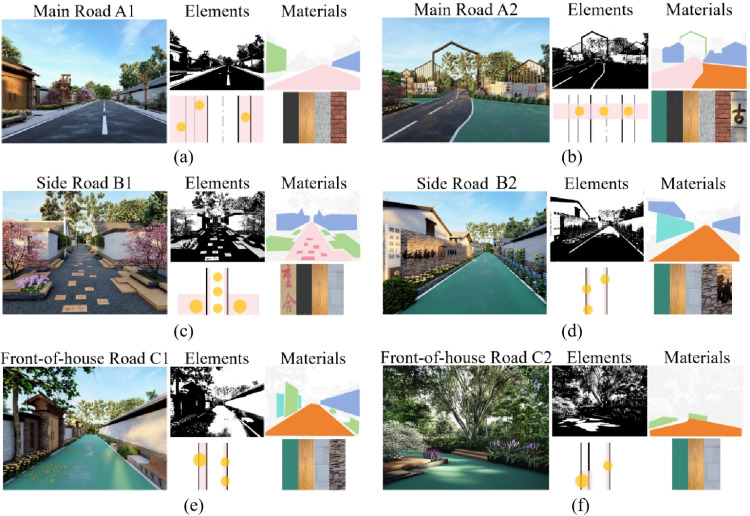
Six design images of three types of street scales. (a) The design rendering of Main Road A1, along with its design elements and materials; (b) The design rendering of Main Road A2, along with its design elements and materials;. (c) The design rendering of Side Road B1, along with its design elements and materials; (d) The design rendering of Side Road B2, along with its design elements and materials; (e) The design rendering of Front-of-house Road C1, along with its design elements and materials; (f) The design rendering of Front-of-house Road C2, along with its design elements and materials.

**Fig 3:**
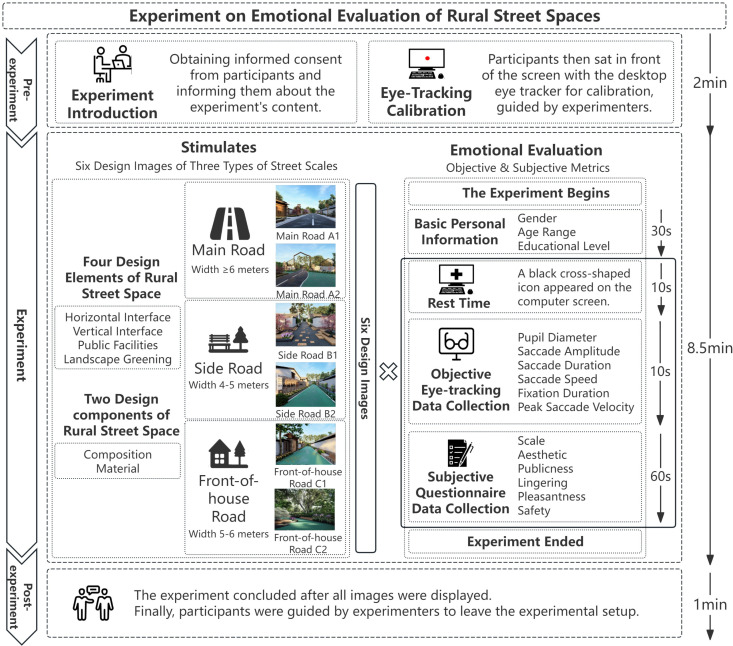
Experiment on emotional evaluation of rural street spaces.

We observed that different design elements within street space influence residents’ public activity behaviors. we first selected three representative scales of rural street spaces: main roads, side roads, and front-of-house road, as depicted in Fig 1. Subsequently, we developed different design schemes for these three types of roads, focusing on variations in materials, colors, and landscaping configurations, with two design options created for each scale. To investigate the impact of various design elements of rural street space on participants’ public activities and their preferences for different design elements, we conceptualized different combinations of four street design elements—horizontal interface, vertical interface, public facilities, and landscape greening—within three levels of rural street space: main road (width ≥6 meters), side road (width = 4–5 meters), and front-of-house road (width = 5–6 meters). Considering the existing site conditions and experimental requirements, we constructed six categories of rural street space design styles across three levels (Main Road A1, Main Road A2, Side Road B1, Side Road B2, Front-of-house Road C1, Front-of-house Road C2), as shown in Table 1. A total of six basic experimental stimulus images were generated, aiming to replicate authentic rural street space using SketchUp and Lumion modeling techniques, combined with actual site photographs, as depicted in Fig 2. Finally, an eye-tracking experiment was conducted to analyze people’s preferences for the various design schemes, as depicted in Fig 3.

**Table 1:** Constituent elements and materials of rural street space design.

Design Images		Horizontal Interface	Vertical Interface	Public Facilities	Landscape Greening
**Main Road A1**	**Composition**	Middle roadway for vehicular traffic Sidewalks on both sides	Building entrances on the left Building walls on both sides	Benches, lamp posts and flower beds on both sides	Flower beds with water features and trees of various sizes on both sides
	**Materials**	Black asphalt and white granite road surface	Wooden building entrances White granite and red brick walls	Wooden benches and lamp posts Black stone base Wooden flower beds	Yellow-green flowers Red-white-green trees
**Main Road A2**	**Composition**	Middle roadway for vehicular traffic Mixed pedestrian and vehicular traffic on both sides	Central entrance signage and landscape wall	None	Flower trees on the left Shrubs surrounding the landscape wall Trees behind the landscape wall
	**Materials**	Black asphalt road surface Green plastic road surface	White stone and red brick landscape wall Black text slogans Wooden grilles	None	Yellow flowers Red-green small trees Green shrub trees
**Side Road B1**	**Composition**	Mixed pedestrian and vehicle traffic roads	Building enclosure walls on both sides	Seating and flower Beds on both sides	Flower shrubs Large trees at the back
	**Materials**	Black asphalt road surface Yellow relief brick pavers	White granite enclosure wall Red brick enclosure wall	Wooden fixed seats Black stone flower beds	Red and purple flower Green bamboo trees
**Side Road B2**	**Composition**	Mixed pedestrian and vehicle traffic roads	Walls on both sides of the buildings Left side scenic wall	Flower beds Directional signboard on the left Streetlights	Flowers on both sides Bushes on the left Large trees in the back
	**Materials**	Green plastic road surface	White granite, red brick, scenic wall Black text signage Wooden window decoration	Black stone flower bed Stone and wooden directional signboard Wooden streetlights	Yellow and purple flowers Green bushes and trees
**Front-of-house Road** **C1**	**Composition**	Mixed pedestrian and vehicle traffic roads	Left-side building entrance Walls on both sides of the building	Flowerbeds on both sides Flowerbed with seating on the right side	Flowers on the left side Shrubs on the right side Large trees on the left rear side
	**Materials**	Green plastic road surface	Wooden building entrance White granite wall red brick wall	Wooden benches Concrete benches	Yellow flowers Green shrubs and trees
**Front-of-house Road** **C2**	**Composition**	Mixed pedestrian and vehicle traffic roads	None	Seats on both sides Planters on both sides	Flowers, shrubs, and trees
	**Materials**	Green plastic road surface	None	Wooden seats Black stone planters	Purple and red flowers Green shrubs and trees White small trees

### 2.2. Data collection

#### 2.2.1. Overall spatial perception.

The design elements of rural street space influence various aspects such as the perception of scale, aesthetics, publicness, linger time, enjoyment, and sense of security [36]. These factors have an impact on the occurrence and extent of different public space activities. In this study, we first examined and compared participants’ subjective perceptions of scale, aesthetics, publicness, linger time, enjoyment, and sense of security regarding six different designs of rural street space. We identified the design elements that affect the overall perception of rural street space. Next, we analyzed the influence of the six types of rural street space designs on eye-tracking data. Finally, combining gaze preference analysis, we identified the design elements that were most attractive to participants.

The experimental data mainly consist of eye-tracking data and questionnaire survey data collected from participants observing different street public space environments.

#### 2.2.2. Eye-tracking data.

Eye-tracking data was captured using the aSee Pro desktop eye-tracker to monitor participants’ gaze patterns during street scene preference selection and behavioral experiments. The sampling rate was set at 140 Hz, with participants viewing each image for 10 seconds. We focused on six eye-tracking metrics, Average Pupil Diameter (APD): Indicates the level of relaxation and alertness participants experience towards the design of the constituent elements. A larger average pupil diameter suggests higher alertness, while a smaller diameter indicates higher relaxation [37]. Average Saccade Amplitude, Average Saccade Duration, Peak Saccade Velocity, and Average Saccade Velocity (APR): Used to evaluate the range of information participants acquire and reflect the salient features of the objects. Average Fixation Duration (AFD): Indicates participants’ preferences for street interface elements. A longer fixation time suggests greater interest in the fixated element [38]. Additionally, Area of Interest (AOI) segmentation analysis was employed to examine revisit frequency, average pupil diameter, and initial fixation duration across different regions of the image. Revisit frequency indicates the level of interest participants have in the constituent elements; a higher revisit frequency suggests greater interest in that design element. Initial fixation duration reflects the duration of participants’ first attention to the design element [39].

#### 2.2.3. Questionnaire data.

The questionnaire included evaluations of participants’ perceptions of scale, aesthetics, publicness, linger time, enjoyment, and sense of security regarding the street space, rated on a scale of “5, 4, 3, 2, 1,” representing “excellent, good, fair, poor, very poor” respectively, as shown in Table 2.

**Table 2:** Subjective questionnaire questions and indicators.

Question: Please answer the following questionnaire based on the picture you just saw, the questionnaire included evaluations of your perceptions of scale, aesthetics, publicness, lingering, enjoyment, and sense of security regarding the street space, rated on a scale of “5, 4, 3, 2, 1,” representing “excellent, good, fair, poor, very poor” respectively.	
Scale	Appropriate scale ○5 ○4 ○3 ○2 ○1 Inappropriate scale
Aesthetics	Beautiful ○5 ○4 ○3 ○2 ○1 Ugly
Publicness	Public ○5 ○4 ○3 ○2 ○1 Private
Lingering	Pause and linger ○5 ○4 ○3 ○2 ○1 Walk briskly through
Enjoyment	Feel enjoyable ○5 ○4 ○3 ○2 ○1 Feel unenjoyable
Security	Feel safe ○5 ○4 ○3 ○2 ○1 Feel unsafe

### 2.3. Experimental procedure

We conducted eye-tracking experiments between December 7 and December 21, 2023. The experiment was conducted in a separate enclosed laboratory, ensuring constant lighting, temperature, and humidity conditions throughout the process. The experiment utilized the aSee Pro remote eye tracker for eye-tracking analysis. The eye tracker was fixed below a 27-inch LCD display with a resolution of 1920 × 1080. The accompanying aSee Studio software was installed on a desktop computer equipped with an NVIDIA GTX 1060 graphics card and running the Windows 10 Pro 64-bit operating system. The experiment consisted of three stages: pre-experiment, experiment, and post-experiment. During the pre-experiment stage, preparations were made, including obtaining informed consent from participants and informing them about the experiment’s content. Participants then sat in front of the screen with the desktop eye tracker for calibration, guided by experimenters. The basic experimental procedure was introduced by the experimenters. In the experiment stage, participants began the experiment as instructed. At the beginning of the experiment, the words “The experiment begins” appeared on the screen. Subsequently, a questionnaire collecting basic personal information—including gender, age range, and educational level—was presented. Participants had 30 seconds to use the mouse to select their answers to the three questionnaire items. Following this, the participants viewed six rendered images of street space design, with each image displayed for 10 seconds. Before viewing each image, there was a 10-second eye rest period during which a black cross-shaped icon appeared on the computer screen. This corrects drift caused by head movements, ensures a fixed starting position, and mitigates potential residual effects [40]. After each image, participants had 60 seconds to complete the corresponding subjective evaluation questionnaire. Eye-tracking data of participants were continuously recorded during the process of viewing the street space design images. The experiment concluded after all images were displayed, the words ‘Experiment ended’ appeared on the computer screen. Finally, participants were guided by experimenters to leave the experimental setup. The entire procedure, from the beginning of the experiment to its conclusion, lasted approximately 11 and a half minutes.

### 2.4. Participants

To obtain more genuine and objective reactions from participants regarding their initial impressions of the experimental photos, between December 1 and December 7, 2023, we recruited 33 participants who had never lived in the village before, instead of selecting local villagers. In previous studies, sample sizes have varied from 20 [41], 30 [42], to 40 [37] participants. Therefore, selecting 33 participants ensures sufficient representativeness and reliability. Local villagers, having resided in the village for an extended period, develop personal preferences toward the street spaces they use daily. Additionally, repeated exposure to familiar spatial scenes may lead to visual fatigue. Therefore, to ensure the objectivity of the eye-tracking experiment data and meet the experimental requirement of first-time stimulus viewing, we selected participants who had not previously lived in the village as subjects for the study. The selection criteria for participants during the preliminary recruitment phase were as follows: educational background is at least high school level or higher, aged between 18 and 60 years old(mean = 27.3, SD = 9.8) with basic reading and comprehension abilities; no significant visual impairments, with nearsightedness of up to 800 degrees and no astigmatism; and a roughly equal gender ratio of approximately 1:1.

We provided the participants with a detailed explanation of the experimental procedure and the subsequent purpose of collecting eye-tracking data, and obtained verbal informed consent from all participants. Since the informed consent form is presented after the participants enter the laboratory and before the experiment begins, to prevent excessive visual fixation from causing visual fatigue and affecting the accuracy of the research data, we conducted a verbal reading of the informed consent form before the experiment. Participants were asked to give verbal consent, and the entire process was monitored and recorded on video. This method of obtaining verbal informed consent was approved by the Institutional Review Board (IRB). This study was approved by the Biomedical Ethics Committee of the Medical College of Hebei University of Engineering (Approval No.: BER-YXY-2023031, Validity Period: 2023-06-10 to 2023-12-30). The study adheres to the ethical standards outlined in the Declaration of Helsinki. The recruitment period lasted from December 1 to December 7, 2023.

### 2.5. Data analysis

In this study, we first conducted preliminary processing of the eye-tracking and questionnaire data using Python to remove outliers. The reliability of the subjective questionnaire data was tested using Cronbach’s alpha. If Cronbach’s alpha exceeds the threshold of 0.8, the data is considered reliable and valid. Then, we used SPSS to perform normality tests on the data. For data that followed a normal distribution, we conducted ANOVA analysis, while for data that did not follow a normal distribution, we conducted non-parametric analysis. We used Origin 2022 for the analysis and visualization of eye-tracking data and subjective questionnaire data. The eye-tracking data and subjective questionnaire data for the three street scales were analyzed through comparative analysis. For data showing significant differences (p ≤ 0.05), a post-hoc test was conducted based on variations in materials and composition to identify the street scales, compositions, and materials that influence corresponding emotional responses. Additionally, gaze heatmaps and Areas of Interest (AOI) for the six street images were analyzed to comprehensively examine visual preferences. Finally, we performed Pearson correlation analysis on the questionnaire data and eye-tracking data to explore the relationship between the two. By integrating subjective questionnaire data with objective eye-tracking data, we analyzed the street space design elements that influence people’s emotional responses. This comprehensive approach allows for identifying key spatial characteristics, materials, and compositions that contribute to emotional perception and user experience in street environments.

## 3. Results

Upon completion of the experiment, data were screened for validity based on eye-tracking capture rates exceeding 80% [38]. This criterion resulted in a final set of 31 valid datasets.

### 3.1. The impact of rural street scale on spatial perception

#### 3.1.1. Analysis of subjective questionnaire perception data.

Firstly, the reliability of the subjective questionnaire data was assessed using SPSS. The reliability test yielded a Cronbach’s alpha value of 0.826, which exceeds the threshold of 0.8. This indicates that the data is reliable and internally consistent, providing confidence in the validity of the questionnaire responses, as shown in Table 3.

**Table 3:** Reliability statistics.

Cronbach’s Alpha	Number of items
0.826	6

To understand the impact of different scales of rural street spaces on participants’ subjective perceptions, we employed Kruskal-Wallis tests (H-tests) to compare the perceptions of space scale, aesthetics, publicness, lingering duration, enjoyment, and safety among main roads, side roads, and front roads.

**Fig 4:**
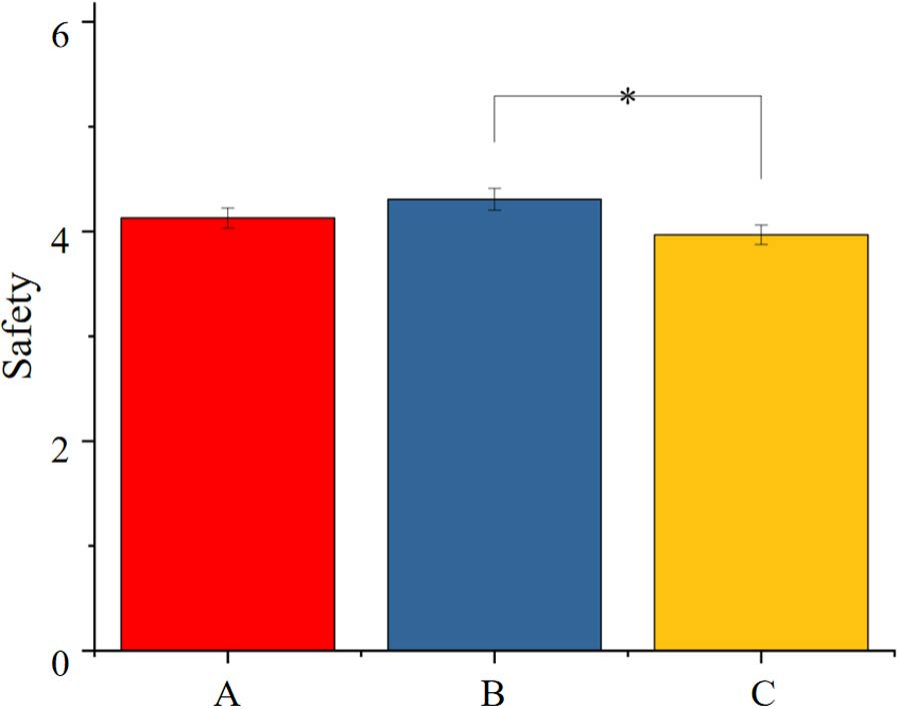
Comparison of safety across three scales of rural streets. (A: Main Road; B: Side Road; C: Front-of-house Road).

The results revealed a significant difference in safety among the three scales of rural streets (p = 0.015), which is below 0.05, as shown in Table 4. Post-hoc comparisons indicated a significant difference in safety perception between side roads and front roads, with side roads showing significantly higher safety perception. However, there were no significant differences observed in spatial scale, aesthetics, publicness, lingering duration, and enjoyment among the three scales of rural streets, indicating that the scale of rural streets does not significantly influence the aforementioned spatial perceptions, as shown in Fig 4.

**Table 4:** The H-test results for subjective questionnaire perception data on rural street space of different scales.

	Scale	Aesthetic	Publicness	Lingering	Pleasantness	Safety
**Kruskal-Wallis H**	1.216	2.859	2.416	0.197	5.058	8.378
**p-value**	0.545	0.239	0.299	0.906	0.080	0.015

#### 3.1.2. Analysis of objective eye-tracking data.

First, the normality of the eye-tracking data sets including average pupil diameter, average saccade amplitude, average saccade velocity, and average fixation duration were examined using the Shapiro-Wilk test in SPSS. For the normally distributed data, one-way analysis of variance (ANOVA) was conducted, whereas non-parametric tests were employed for non-normally distributed data. As shown in Table 5, results revealed that different scales of rural street dimensions significantly influenced participants’ average pupil diameter, average saccade amplitude, and average saccade velocity (p < 0.05). However, there was no significant effect on participants’ average saccade duration, average fixation duration and peak saccade velocity (p > 0.05).

**Table 5:** Comparative analysis of objective eye-tracking data across different scales of rural street dimensions.

	Average Pupil Diameter	Average Saccade Amplitude	Average Saccade Duration	Average Saccade Velocity	Average Fixation Duration	Peak Saccade Velocity
**p-value**	0.043	0.030	0.475	0.036	0.066	0.593

**Fig 5:**
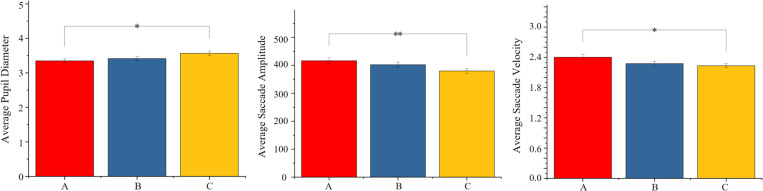
Comparison of eye-tracking data across three scales of rural street spaces. (A: Main Road; B: Side Road; C: Front-of-house Road).

The post-hoc comparison analysis reveals significant differences primarily between the main road and the front yard road, as shown in Fig 5. (1) The average pupil diameter of the front yard road is significantly larger than that of the main road, indicating that the width of 5–6 meters in the front yard road is more visually appealing. (2) The average eye blink amplitude and speed on the main road are significantly greater than those on the front yard road, suggesting that in spaces with widths greater than 6 meters and separate lanes for pedestrians and vehicles, people acquire visual information at a faster pace, making the spatial characteristics more distinct. Therefore, the main road and the front yard road can be regarded as focal points for visual perception design.

### 3.2. Impact of rural street design elements on spatial perception

In order to investigate the influence of different rural street spatial design elements on subjects’ subjective perceptions, we utilized LSD analysis to compare and analyze the spatial scale perception, aesthetics, publicness, linger duration, pleasantness, and safety of six rural street spatial designs under the influence of four design elements, as shown in Table 6.

**Table 6:** Comparative analysis of subjective perception across different design elements and materials.

			Scale	Aesthetic	Publicness	Lingering	Pleasantness	Safety
**Horizontal Interface**	**Composition**	**p-value**	0.093	0.122	0.179	0.168	0.409	0.959
		**Grouping variables**	1. Middle roadway for vehicles with pedestrian sidewalks on both sides. 2. Middle roadway for vehicles with mixed pedestrian and vehicular traffic on both sides. 3. Roadway for mixed pedestrian and vehicular traffic.					
	**Material**	**p-value**	0.098	0.054	0.013	0.045	0.032	0.004
		**Grouping variables**	1. Black asphalt road surface with white granite pavement. 2. Black asphalt road surface with green rubber pavement. 3. Black asphalt road surface with yellow relief floor tiles. 4. Green rubber pavement.					
**Vertical** **Interface**	**Composition**	**p-value**	0.295	0.047	0.013	0.013	0.018	0.008
		**Grouping variables**	1. Building entrance with architectural enclosure. 2. Central entrance sign, scenic wall. 3. Architectural enclosure. 4. Architectural enclosure, scenic wall. 5. None.					
	**Material**	**p-value**	0.293	0.350	0.014	0.026	0.018	0.008
		**Grouping variables**	1. Wood, white granite, red brick. 2. White granite, red brick, black stone text, wood. 3. White granite, red brick. 4. None.					
**Public** **Facilities**	**Composition**	**p-value**	0.098	0.047	0.049	0.032	0.426	0.947
		**Grouping variables**	1. Seats, streetlights, flower beds. 2. None. 3. Seats, flower beds. 4. Streetlights, flower beds, signage.					
	**Material**	**p-value**	0.312	0.046	0.002	0.038	0.288	0.097
		**Grouping variables**	1. Wood, black stone. 2. None. 3. Wood, black stone, white stone. 4. Wood, concrete.					
**Landscape Greening**	**Composition**	**p-value**	0.093	0.208	0.039	0.046	0.026	0.005
		**Grouping variables**	1. Flowers, water features, trees. 2. Flowers, trees, shrubs. 3. Flowers, trees.					
	**Material**	**p-value**	0.099	0.047	0.002	0.013	0.034	0.005
		**Grouping variables**	1. Yellow, red, white, green. 2.Yellow, red, green. 3.Red, purple, green. 4.Yellow, purple, green. 5.Yellow, green. 6.Red, purple, white, green.					

The results indicate significant differences among various design elements in terms of spatial aesthetics, public utility, dwell time, enjoyment, and perceived safety. Specifically, (1) different components of vertical interface, public facilities composition, and material, as well as the material colors of landscape greening, exhibit significant differences in spatial aesthetics; (2) the material of horizontal interface, in conjunction with the composition and material of vertical interface, public facilities, and landscape greening, significantly influences spatial public utility and dwell time; (3) the material of different horizontal interfaces, along with the composition and material colors of vertical interface and landscape greening, demonstrate significant differences in spatial enjoyment and perceived safety.

**Fig 6:**
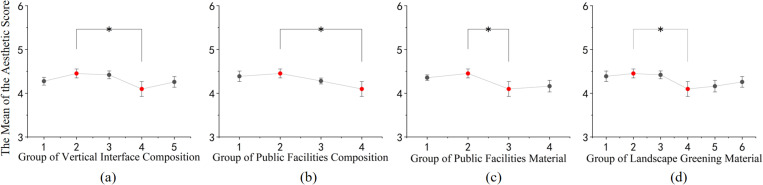
Post-hoc comparisons were conducted to examine the differences in aesthetic appeal among various compositions and materials in rural street space. (a) Post-hoc comparison results of the aesthetic evaluation of vertical interface composition; (b) Post-hoc comparison results of the aesthetic evaluation of public facilities composition; (c) Post-hoc comparison results of the aesthetic evaluation of public facilities material; (d) Post-hoc comparison results of the aesthetic evaluation of landscape greening material. (* p<=0.05, ** p<=0.01, *** p<=0.001).

The post-hoc comparisons reveal that, as shown in Fig 6, concerning aesthetic appeal: (1) Among the structural elements, the middle entrance signage and the landscape wall exhibit significantly higher aesthetic appeal compared to the surrounding architectural walls and landscape walls; the type and quantity of public facilities do not significantly impact aesthetic appeal. (2) Regarding material composition, landscapes with red-colored plants exhibit higher aesthetic appeal; the material of public facilities does not significantly affect aesthetic appeal.

**Fig 7:**
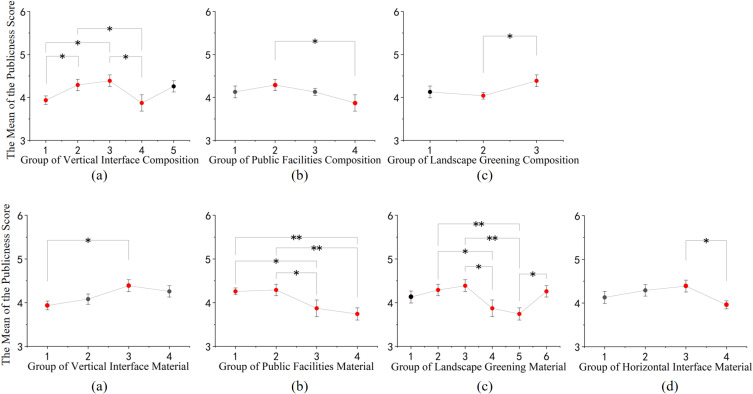
Post-hoc comparison of different rural street space configurations and materials in terms of publicness. (a) Post-hoc comparison results of the publicness evaluation of vertical interface composition; (b) Post-hoc comparison results of the publicness evaluation of public facilities composition; (c) Post-hoc comparison results of the publicness evaluation of landscape greening composition; (d) Post-hoc comparison results of the publicness evaluation of vertical interface material; (e) Post-hoc comparison results of the publicness evaluation of public facilities material; (f) Post-hoc comparison results of the publicness evaluation of landscape greening material; (g) Post-hoc comparison results of the publicness evaluation of horizontal interface material. (* p<=0.05, ** p<=0.01, *** p<=0.001).

In terms of publicness, as shown in Fig 7: (1) Among the constituent elements, the vertical interface with a central entrance sign exhibits better publicness when adorned with flowers and trees. (2) Regarding the materials of the elements, combinations such as black asphalt with yellow relief bricks, vertical interfaces combining white granite with red bricks, public facilities featuring wood combined with black stone, and spaces adorned with red and green landscape plants tend to score higher in publicness.

**Fig 8:**
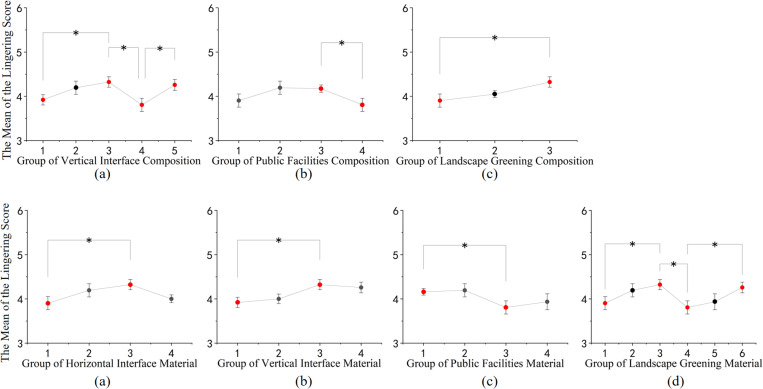
Post-hoc comparison of different rural street space configurations and materials on lingering. (a) Post-hoc comparison results of the lingering evaluation of vertical interface composition; (b) Post-hoc comparison results of the lingering evaluation of public facilities composition; (c) Post-hoc comparison results of the lingering evaluation of landscape greening composition; (d) Post-hoc comparison results of the lingering evaluation of horizontal interface material; (e) Post-hoc comparison results of the lingering evaluation of vertical interface material; (f) Post-hoc comparison results of the lingering evaluation of public facilities material; (g) Post-hoc comparison results of the lingering evaluation of landscape greening material. (* p<=0.05, ** p<=0.01, *** p<=0.001).

In terms of dwell time within the spatial context, as shown in Fig 8: (1) Among the constitutive elements, spaces characterized by fewer and more open elements in the vertical interface, along with amenities such as seating and flower beds within the public facilities, and the combination of flowers and trees within the landscape greenery, tend to encourage people to linger within the space. (2) Regarding material composition, combinations such as black asphalt and yellow relief tiles for ground materials, white granite and red brick for vertical interface materials, wood and black stone for public facility materials, and landscape greenery spaces with a rich combination of red and green hues, tend to foster prolonged stays within the space.

**Fig 9:**
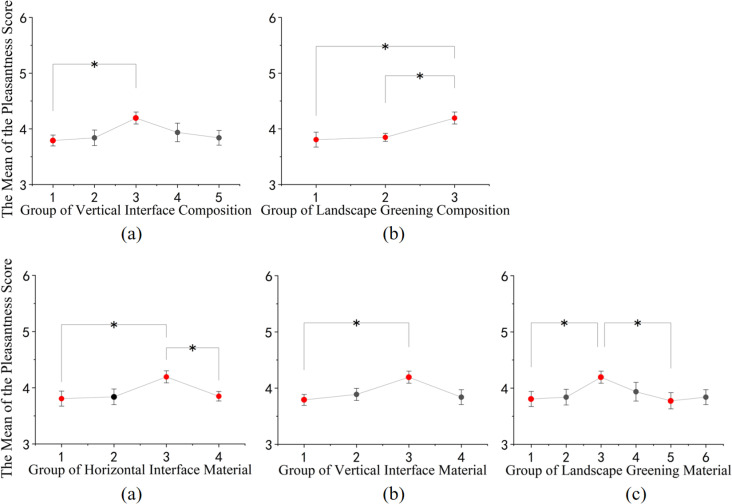
Post-hoc comparison of the impact of different rural street space compositions and materials on pleasantness. (a) Post-hoc comparison results of the pleasantness evaluation of vertical interface composition; (b) Post-hoc comparison results of the pleasantness evaluation of landscape greening composition; (c) Post-hoc comparison results of the pleasantness evaluation of horizontal interface material; (d) Post-hoc comparison results of the pleasantness evaluation of vertical interface material; (e) Post-hoc comparison results of the pleasantness evaluation of landscape greening material. (* p<=0.05, ** p<=0.01, *** p<=0.001).

In terms of spatial enjoyment, as shown in Fig 9: (1) In terms of compositional elements, street spaces with fewer vertical structural elements and higher spatial openness, as well as those composed of floral and arboreal elements in landscape greenery, tend to yield higher levels of spatial enjoyment. (2) Regarding material composition, street spaces with a combination of black asphalt and yellow relief tiles for horizontal surfaces, white granite and red brick for vertical surfaces, and a mix of red, purple, and green hues in landscape greenery, tend to evoke higher levels of spatial enjoyment among individuals.

**Fig 10:**
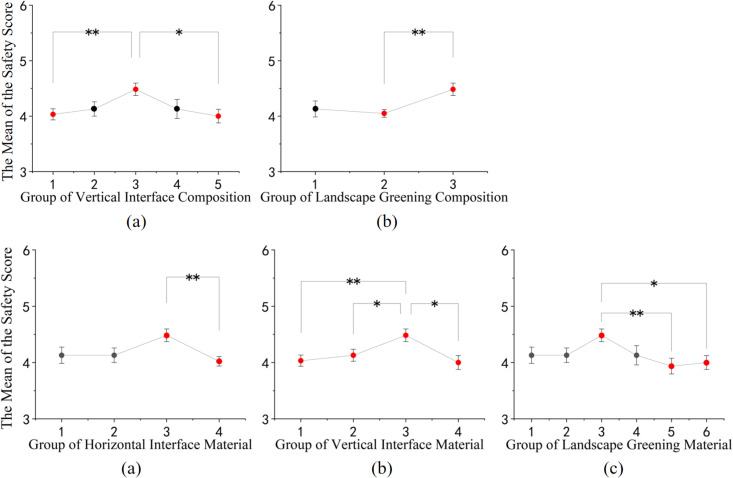
Post-hoc comparison of different rural street space configurations and materials on safety perception. (a) Post-hoc comparison results of the safety evaluation of vertical interface composition; (b) Post-hoc comparison results of the safety evaluation of landscape greening composition; (c) Post-hoc comparison results of the safety evaluation of horizontal interface material; (d) Post-hoc comparison results of the safety evaluation of vertical interface material; (e) Post-hoc comparison results of the safety evaluation of landscape greening material. (* p<=0.05, ** p<=0.01, *** p<=0.001).

In terms of spatial safety perception, as shown in Fig 10: (1) Among the structural elements, semi-enclosed vertical spaces with architectural walls provide a higher sense of safety compared to spaces with elements such as building entrances, landscape walls, and middle entrance signs, as well as fully open spaces. Additionally, street spaces composed of a combination of flowers and trees in landscape greenery tend to offer higher safety perceptions. (2) Regarding material composition, horizontal surfaces featuring a combination of black asphalt and yellow relief tiles, vertical surfaces composed of white granite and red bricks, and street spaces characterized by a combination of red, purple, and green hues in landscape greenery are associated with higher safety perceptions.

### 3.3. Eye movement analysis

#### 3.3.1. Analysis of eye movement data.

To understand the impact of different rural street spatial design elements on participants’ subjective perception, we utilized LSD tests to compare the effects of four design elements on six types of rural street spatial designs. Specifically, we examined the average pupil diameter, average saccade amplitude, average saccade duration, average saccade speed, average fixation duration, and peak saccade velocity. The results are presented in Table 7.

**Table 7:** Comparative analysis of eye movement data across different design elements and materials.

			Average Pupil Diameter	Average Saccade Amplitude	Average Saccade Duration	Average Saccade Velocity	Average Fixation Duration	Peak Saccade Velocity
**Horizontal Interface**	**Composition**	**p-value**	0.036	0.082	0.202	0.005	0.038	0.241
		**Grouping variables**	1. Middle roadway for vehicles with pedestrian sidewalks on both sides. 2. Middle roadway for vehicles with mixed pedestrian and vehicular traffic on both sides. 3. Roadway for mixed pedestrian and vehicular traffic.					
	**Material**	**p-value**	0.030	0.082	0.080	0.009	0.040	0.274
		**Grouping variables**	1. Black asphalt road surface with white granite pavement. 2. Black asphalt road surface with green rubber pavement. 3. Black asphalt road surface with yellow relief floor tiles. 4. Green rubber pavement.					
**Vertical** **Interface**	**Composition**	**p-value**	0.008	0.156	0.081	0.009	0.002	0.170
		**Grouping variables**	1. Building entrance with architectural enclosure. 2. Central entrance sign, scenic wall. 3. Architectural enclosure. 4. Architectural enclosure, scenic wall. 5. None.					
	**Material**	**p-value**	0.009	0.129	0.146	0.029	0.001	0.169
		**Grouping variables**	1. Wood, white granite, red brick. 2. White granite, red brick, black stone text, wood. 3. White granite, red brick. 4. None.					
**Public** **Facilities**	**Composition**	**p-value**	0.018	0.036	0.214	0.002	0.018	0.183
		**Grouping variables**	1. Seats, streetlights, flower beds. 2. None. 3. Seats, flower beds. 4. Streetlights, flower beds, signage.					
	**Material**	**p-value**	0.041	0.036	0.106	0.006	0.006	0.114
		**Grouping variables**	1. Wood, black stone. 2. None. 3. Wood, black stone, white stone. 4. Wood, concrete.					
**Landscape Greening**	**Composition**	**p-value**	0.736	0.162	0.134	0.199	0.332	0.662
		**Grouping variables**	1. Flowers, water features, trees. 2. Flowers, trees, shrubs. 3. Flowers, trees.					
	**Material**	**p-value**	0.008	0.023	0.081	0.006	0.002	0.116
		**Grouping variables**	1. Yellow, red, white, green. 2.Yellow, red, green. 3.Red, purple, green. 4.Yellow, purple, green. 5.Yellow, green. 6.Red, purple, white, green.					

The results indicate significant differences among various design elements in terms of participants’ average pupil diameter, average saccade amplitude, average saccade velocity, and average fixation duration. Specifically: (1) Different compositions and materials of horizontal interfaces, vertical interfaces, public facilities, and the coloration of landscape greenery exhibit significant differences in average pupil diameter, average saccade velocity, and average fixation duration. (2) The composition and materials of public facilities, as well as the coloration of landscape greenery, significantly influence the average saccade amplitude.

**Fig 11:**
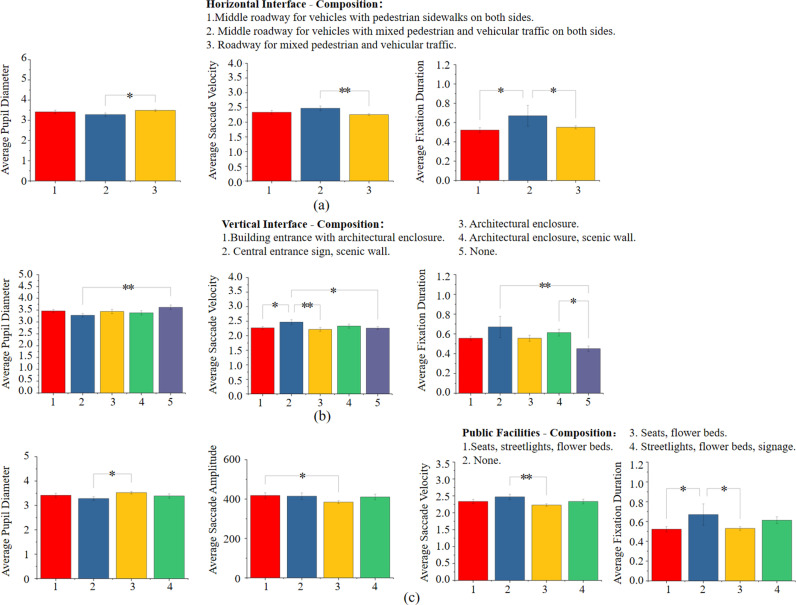
Post-hoc comparisons were conducted to analyze the eye-tracking data among different compositional elements. (a) Post-hoc comparison results of the eye-tracking data of horizontal interface composition; (b) Post-hoc comparison results of the eye-tracking data of vertical interface composition; (c) Post-hoc comparison results of the eye-tracking data of public facilities composition. (* p<=0.05, ** p<=0.01, *** p<=0.001).

The post-hoc comparisons revealed the following insights regarding the compositional elements in rural street spaces, as shown in Fig 11: (1) Horizontal Interface Elements: Among the horizontal interface elements, only the road with mixed pedestrian and vehicular traffic significantly increased the average pupil diameter compared to other elements, indicating heightened visual interest. Roads with mixed pedestrian and vehicular traffic also showed significantly higher average eye movement speed and duration of gaze compared to the other two types of roads, suggesting that the more diverse the road composition elements, the higher the visual attention they attract. (2) Vertical Interface Elements: Spaces without enclosed vertical interfaces resulted in larger pupil diameters, indicating greater visual interest. Vertical elements such as entrance signage and decorated walls, especially those adjacent to buildings with decorative walls, elicited higher average eye movement speed and duration of gaze, signifying increased visual attention. (3) Public Facilities: Street spaces featuring abundant public facilities such as seating, flower beds, streetlights, and directional signs showed relatively higher average pupil diameter, eye movement amplitude, speed, and duration of gaze. This suggests that a greater variety of public facilities tends to capture more visual attention.

**Fig 12:**
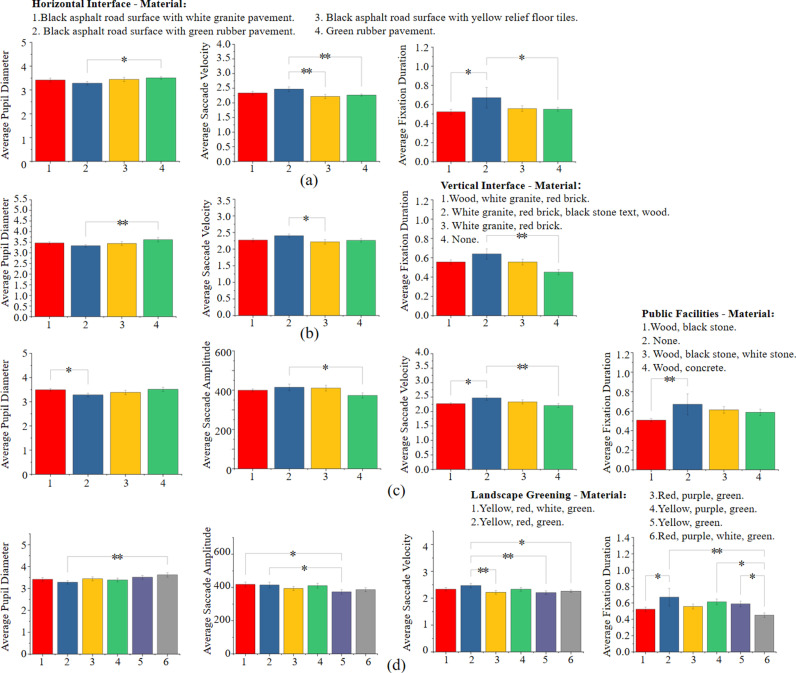
Post-hoc comparisons were conducted to analyze the eye-tracking data among different material textures. (a) Post-hoc comparison results of the eye-tracking data of horizontal interface material; (b) Post-hoc comparison results of the eye-tracking data of vertical interface material; (c) Post-hoc comparison results of the eye-tracking data of public facilities material; (d) Post-hoc comparison results of the eye-tracking data of landscape greening material. (* p<=0.05, ** p<=0.01, *** p<=0.001).

In terms of material texture within the compositional elements, as shown in Fig 12: (1) Horizontal Interface Materials: Green plastic pavement and its combination with black asphalt significantly increased the average pupil diameter, eye movement amplitude, and duration of gaze compared to other materials. This indicates that green plastic pavement is more effective in capturing people’s attention. (2) Vertical Interface Materials: Vertical interfaces decorated with white granite, red brick, black stone text, and wooden embellishments resulted in higher average eye movement amplitude and duration of gaze. This suggests that such materials attract more visual attention. (3) Public Facility Materials: Combinations of wood and black stone in public facilities were more effective in attracting visual attention. (4) Landscape Color Combinations: Spaces with richer landscape color combinations and higher landscape coverage exhibited larger average pupil diameter, eye movement amplitude, and speed, indicating higher interest in the space. Spaces with combinations of red, yellow, green, and purple colors resulted in longer average duration of gaze, indicating increased visual attention.

#### 3.3.2. Analysis of visual preferences.

In the visual preference analysis, we explored the preferences of participants for different design elements of rural streets. By presenting participants with a series of images or scenes representing various design elements and recording their gaze patterns, we aimed to understand the extent of their preferences for different design choices [39].

**Fig 13:**
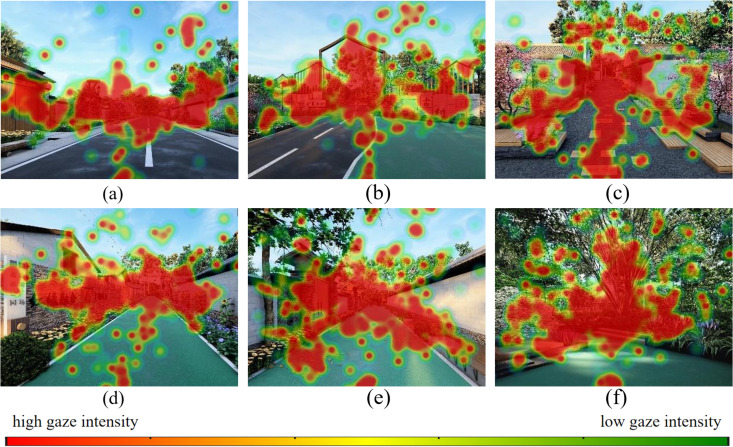
The gaze heatmaps for the six street space design images. (a) The gaze heatmaps for the main road A1; (b) The gaze heatmaps for the main road A2; (c) The gaze heatmaps for the side road B1; (d) The gaze heatmaps for the side road B2; (e) The gaze heatmaps for the front-of-house road C1; (f) The gaze heatmaps for the front-of-house road C2.

The visual preference analysis for the constituent elements reveals the following, as shown in Fig 13: (1) Horizontal Interface: Within the horizontal interface, pedestrian lanes failed to attract visual attention compared to mixed-use lanes, which garnered more visual interest. (2) Vertical Interface: Concerning the vertical interface, elements such as building entrances, entrance signage perpendicular to the line of sight, and decorative walls effectively captured visual attention. (3) Public Facilities: In terms of public facilities, features like seating areas and streetlights emerged as focal points of visual attention. (4) Landscape Greenery: Within landscape greenery, flowers and trees stood out as primary focal points of visual attention.

Regarding the visual preference analysis based on constituent element materials: (1) Horizontal Interface Materials: Visual preferences for horizontal interface materials were generally ranked as follows: yellow relief tiles > green plastic ground > black asphalt pavement. (2) Vertical Interface Materials: Materials such as wood and brick decorations on walls or building entrances garnered stronger visual appeal compared to walls made of white granite. (3) Public Facility Materials: Wooden benches and lamp posts attracted significant visual attention among public facility materials. (4) Landscape Greenery Color Composition: Green plants and flowers in red and purple hues tended to attract more visual attention.

#### 3.3.3. AOI data analysis.

AOI (Area of Interest) data analysis involves examining participants’ gaze patterns within different regions of interest during eye-tracking experiments [43]. Through AOI data analysis, we can gain insights into the extent of participants’ attention to specific areas, allowing for a deeper understanding of their attentional preferences and behavioral patterns [44]. During AOI data analysis, the experimental environment is typically divided into several predefined regions, and data such as the number of fixations and the total fixation duration are recorded for each region. By analyzing and comparing these data, we can determine the level of attention devoted to different regions and analyze the impact of each region on participants’ behavior and cognitive processes. In this study, the six design drawings were divided into an average of 5x5 rectangular grids. By analyzing and comparing the number of revisits, average pupil diameter, and first fixation duration within each grid, we comprehensively analyzed participants’ gaze behavior under different design elements and materials.

**Fig 14:**
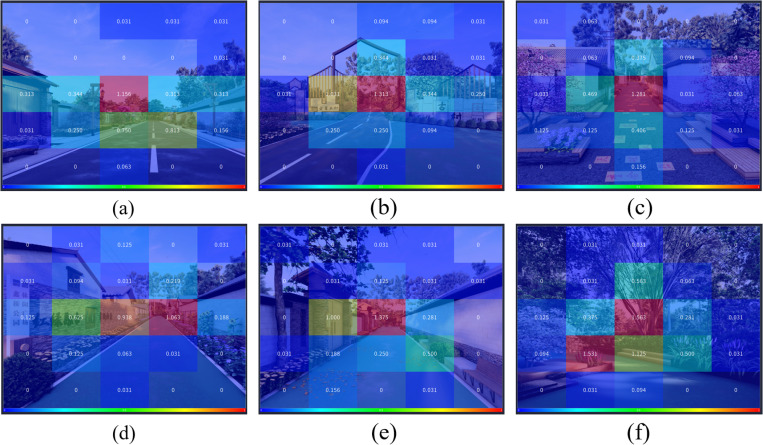
AOI map of revisit counts in the six street space design images. (a) The AOI map of revisit counts in the main road A1; (b) The AOI map of revisit counts in the main road A2; (c) The AOI map of revisit counts in the side road B1; (d) The AOI map of revisit counts in the side road B2; (e) The AOI map of revisit counts in the front-of-house road C1; (f) The AOI map of revisit counts in the front-of-house road C2.

Analysis of the AOI map based on the revisit counts from the six street space design images reveals that elements such as landscape flowers, resting benches, building entrances, landscape walls, and ground relief tiles are among the components that receive relatively higher revisit counts, as shown in Fig 14.

**Fig 15:**
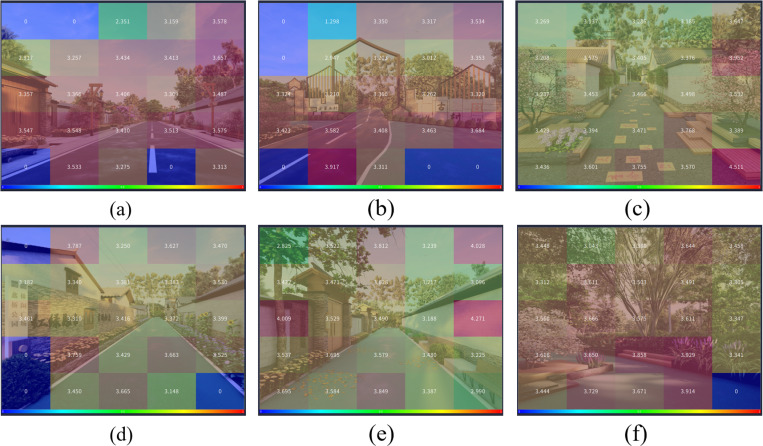
AOI map of average pupil diameter in the six street space design images. (a) The AOI map of average pupil diameter in the main road A1; (b) The AOI map of average pupil diameter in the main road A2; (c) The AOI map of average pupil diameter in the side road B1; (d) The AOI map of average pupil diameter in the side road B2; (e) The AOI map of average pupil diameter in the front-of-house road C1; (f) The AOI map of average pupil diameter in the front-of-house road C2.

Analyzing the AOI data from the six rural street space design images, it can be observed that when participants looked at elements such as flowers, street lamps, directional signs, building entrance gates, green plastic ground surfaces, and seating benches, their average pupil diameter was larger, as shown in Fig 15. This indicates that these design elements and materials are more visually engaging to individuals.

**Fig 16:**
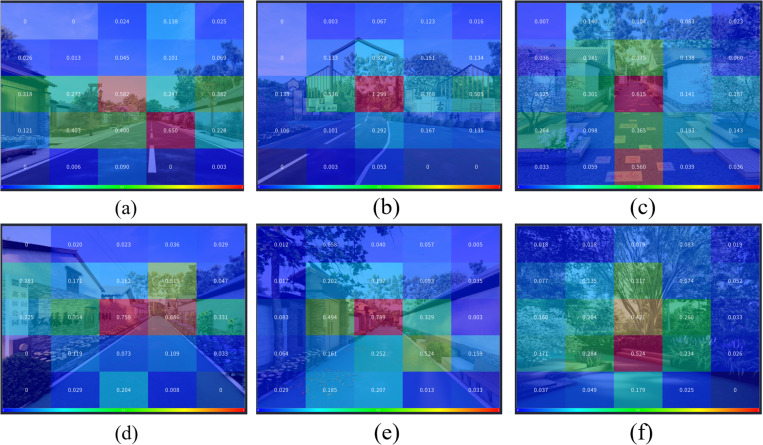
AOI map of first fixation duration in the six street space design images. (a) The AOI map of first fixation duration in the main road A1; (b) The AOI map of first fixation duration in the main road A2; (c) The AOI map of first fixation duration in the side road B1; (d) The AOI map of first fixation duration in the side road B2; (e) The AOI map of first fixation duration in the front-of-house road C1; (f) The AOI map of first fixation duration in the front-of-house road C2.

Analysis of the AOI map based on the first fixation duration reveals that the street space elements and materials that attracted participants’ attention for a longer duration during their first fixation include: streetlights, seating areas, greenery, landscape walls, and yellow relief tiles, as shown in Fig 16.

### 2.4. Correlation analysis

**Fig 17:**
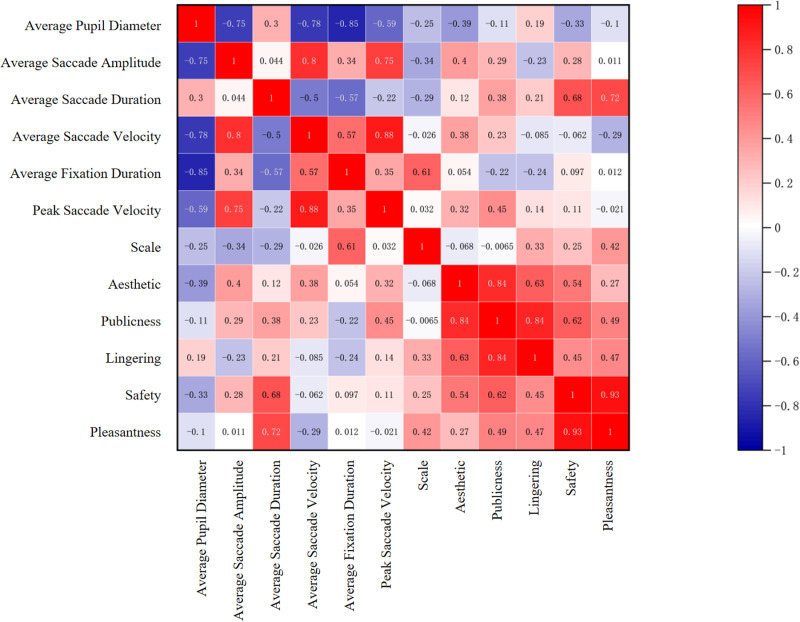
Correlation analysis.

To compare the correlation between subjective questionnaire data and objective eye-tracking data, as well as the correlation between spatial publicness and dwell time with scale perception, aesthetics, pleasantness, and safety, we conducted Pearson correlation analysis using SPSS software on the average values of these two sets of data. The results are shown in the Fig 17 below:

(1) There is a significant positive correlation between spatial scale perception and average gaze duration, with r = 0.61. This suggests that the longer the average gaze duration, the more people perceive the spatial scale as reasonable.
(2) Spatial safety and pleasantness are significantly positively correlated with average saccade duration, with r = 0.68 and 0.72, respectively. This indicates that the longer the average saccade duration, the higher people’s sense of safety and pleasantness in the space.
(3) Spatial publicness is significantly positively correlated with aesthetics (r = 0.84), dwell time (r = 0.84), and safety (r = 0.62). This implies that the more aesthetically pleasing the space, the more people are willing to dwell in it, and the higher the perceived safety, the stronger people’s perception of spatial publicness.
(4) Dwell time is significantly positively correlated with aesthetics (r = 0.63) and spatial publicness (r = 0.84). This suggests that the stronger the aesthetics and publicness of the space, the stronger people’s willingness to dwell in rural street spaces.

## 4. Discussion

This study, based on the influence of different scales and constituent elements, explores the subjective evaluations and behavioral preferences of participants regarding rural street space design. Combined with eye-tracking data and statistical analysis methods, it provides evidence for the positive role of street space design in promoting people’s social interactions and positive spatial experiences in rural areas, thus offering design directions for the construction of healthy and vibrant rural streets.

### 4.1. The influence of spatial scale on safety perception and visual perception

In terms of the influence of rural street spatial scale on safety perception, our research findings indicate that narrower streets tend to make people feel safer in the space. The safety perception is higher in lanes with a width of 4–5 meters compared to main roads and front-yard roads with a width of over 5 meters. Our study found that high walls on both sides of narrow streets may provide a sense of privacy and protection, which aligns with the findings of Gehl (2007) [45] and Mehta (2018) [46]. They suggested that smaller street scales enhance the sense of security by fostering increased community interaction. However, this contrasts with the findings of Nasar and Fisher (1993) [47], who observed that narrow streets in urban environments could heighten the fear of crime. This discrepancy may reflect the distinct cultural and community dynamics between rural and urban settings. In northern rural China, where community ties are strong and streets are primarily used by local residents, high walls may be perceived as an extension of private space, thereby enhancing the sense of security. This perspective is also supported by Newman’s (1972) [48] concept of defensible space, which suggests that enclosed spaces with clear boundaries contribute to a heightened sense of safety. Meanwhile, narrow streets with high walls on both sides give people a stronger sense of safety, while wider roads make people feel more uneasy. Wider streets may induce discomfort due to a sense of exposure. Ewing and Handy (2009) [4] pointed out that in urban settings, wider streets may feel less secure due to reduced community surveillance, a notion that may also be applicable to the context of rural main roads.

Regarding the influence of rural street spatial scale on visual perception, our research findings are as follows: First, main roads and front- of-house roads with a width of over 5 meters are focal points of visual attention. Kang Jian et al. (2018) [49] found a positive correlation between the width-to-height ratio of streets and visual attention comfort, suggesting that wider streets are more likely to attract visual attention and enhance perceived comfort. Second, front-of-house road, people have significantly larger pupil diameter data, indicating greater visual interest. This is consistent with the findings of Beatty and Lucero-Wagoner (2012) [50], who suggested that pupil dilation reflects visual interest or arousal, which may be attributed to the presence of more attention-grabbing features in front yard spaces. Similarly, Ma et al. (2021) [51] support the notion that street design influences the allocation of visual attention, Liu et al. (2025) [52] found that incorporating visual stimuli such as greenery and building facades into street space design can enhance visual attractiveness, indicating that front yard spaces may possess greater visual appeal. Third, on main roads, people exhibit larger saccade amplitudes and saccade speeds, indicating better expression of spatial characteristics. This is consistent with the findings of Simpson and Sellen (2018) [8], who suggested that wider streets facilitate broader visual exploration, requiring larger eye movements to process the surrounding environment.

### 4.2. The influence of design elements and materials on subjective perception

Regarding the influence of spatial design elements in rural streets on subjective perception, our research findings indicate that streets with vertical signage and landscape walls are perceived as more aesthetically pleasing. Spaces with more trees and flowers enhance the publicness of street spaces. Spaces with more rest seats, flower beds, and lower enclosure on vertical surfaces are more open, and people are more likely to linger in these spaces, feeling more joyful. The richer the constituent elements in the space, the safer people feel. Consistent with these findings, Fu et al. (2022) conducted a study on rural community streets, revealing that semi-enclosed natural streets—typically featuring trees and flowers—are perceived as having greater therapeutic potential. This aligns with the perspective that enriched elements enhance feelings of safety and happiness, as more complex and natural environments are associated with positive subjective perception [31]. Furthermore, the study by Park and Garcia (2020) demonstrated that enhanced street lighting, increased street morphological complexity, and diverse activities can promote a sense of security and willingness to stay. This further supports the idea that additional design elements, such as seating areas and flower beds, positively influence perceived safety and prolong the duration of stay [53].

Regarding the influence of material choices in rural street spatial design on subjective perception, our research findings indicate that landscapes with a combination of red and green more aesthetically pleasing. For materials, people perceive spaces with black asphalt and yellow relief bricks on the ground, white granite and red bricks on vertical surfaces, and wood combined with black stone materials in public facilities as more public-friendly, making them more willing to linger in these spaces and feeling more joyful and secure. Although there is limited direct research on rural street materials, Jaglarz (2023) studied urban and architectural spaces and found that the visual quality of materials significantly influences subjective perception, such as attractiveness and a sense of calm. This supports the idea that specific material combinations may enhance the aesthetic appeal and emotional response of rural streets through visual attractiveness [54]. Yu et al. (2024) found that color complexity and harmony are associated with residents’ emotional responses, further supporting the crucial role of material color in shaping subjective perception [55]. Additionally, Seckler et al. (2015) found that specific color combinations, such as red and green, may create a balanced and pleasant visual experience due to their associations with energy and nature [56].

### 4.3. The influence of design elements and materials on visual perception and visual preferences

Regarding the influence of spatial design elements in rural streets on visual perception and preference, our research findings indicate that roads with mixed pedestrian and vehicle traffic, rich public facilities such as seats, flower beds, streetlights, and directional signs, and streets with lower enclosure are more likely to attract people’s visual attention. Similarly, Simpson and Sellen (2018), using mobile eye-tracking, found that complex urban street edges—including signage and buildings—attracted more visual attention [8], supporting the role of intricate design elements in capturing attention. People are more inclined to focus on vertical signage, landscape walls, building entrances, rest seats, streetlights, trees, and flowers. Simpson and Sellen (2019) found that functional and decorative elements, such as seating areas and flower beds, occupy a significant position in pedestrians’ visual attention [57], supporting their role in enhancing public engagement and the willingness to linger.

Regarding the influence of material usage in rural street spatial design on visual perception, our research findings indicate that green plastic roads, white granite, red bricks, black stones, wood, and rich-colored landscape combinations are more likely to attract people’s visual attention and interest. Noland et al. (2017) found that the visual quality of materials significantly influences subjective perception, such as attractiveness and a sense of tranquility [58], supporting the idea that specific material combinations can enhance the aesthetic appeal and emotional response of rural streets through visual attraction.

Regarding the influence of material usage in rural street spatial design on visual preference, our research findings indicate that visual preferences for material colors are as follows: yellow relief bricks > green plastic ground > black asphalt road surface; wood > red brick > white granite; greenery > red > purple. Consistent with these preferences and studies on color perception, specific color combinations, such as green and red, are considered more attractive due to their symbolic associations with nature and energy [54]. These elements and materials are the most interesting to people, leading to repeated and prolonged visual attention. Previous eye-tracking studies have confirmed that elements of interest or those rich in information typically receive longer fixation durations [31] and greater visual attention [57], supporting our findings.

### 4.4. Eye-tracking data reflects subjective perception

Regarding whether eye-tracking data can reflect subjective perception, we found that the longer the average gaze duration on street space, the more satisfied people are with the spatial scale’s reasonableness. Simpson et al. (2019) found that pedestrians tend to focus more on the ground level rather than the upper levels of both pedestrian and non-pedestrian streets, especially on the walking side of non-pedestrian streets [57]. This suggests that the design of the ground level may attract longer gaze durations, influencing the perception of spatial scale. Similarly, the longer the average saccade duration, the more satisfied people are with the space’s pleasantness and safety. Simpson et al. (2019) found that optional activities (e.g., cafés, shops) attract more visual attention than necessary activities (e.g., ATMs, bus stops) [57]. This suggests that engaging elements may enhance the pleasantness of a space, as people tend to scan and focus more on interesting features, potentially improving perceived comfort. In terms of color perception, Wang et al. (2024) found that blue-green color combinations reduce visual stress, creating a calming environment, while purple combinations received higher ratings in both visual perception and subjective evaluation [59]. This may enhance the aesthetic appeal of street spaces, indirectly influencing perceptions of safety and public friendliness.

Meanwhile, aesthetic and safety of rural street space reflect publicness and dwell time. Our research found that spaces more attractive and safer are more inclined to perceive them as more public-friendly and to linger in them. Sheng et al. (2025) found that children exhibited significant visual attention toward miniature vehicles, people, plants, and grass, with these elements attracting more gazes on primary and secondary roads. This suggests that such features may enhance the appeal of street spaces, create a greater sense of safety, and encourage longer dwell times, particularly in child-friendly designs [60]. Wang et al. (2024) further revealed that appropriate brightness contrast has a greater impact on visual quality than hue contrast [59]. These studies collectively support the influence of street design elements on subjective perception, providing a more comprehensive perspective for future research and rural street space design—especially in improving public friendliness and dwell time.

## 5. Conclusions

This study investigates the emotional and behavioral responses of individuals to rural street space design, emphasizing the effects of spatial scale, design elements, and material composition. The integration of subjective evaluation, eye-tracking data, and statistical analysis provides a comprehensive understanding of how rural street design can foster positive spatial experiences and social interactions, contributing to the creation of healthier and more vibrant rural environments.

(1) Spatial scale and spatial perception

Narrow streets, particularly those with widths of 4–5 meters, enhance the perception of safety, while wider roads may evoke unease. Spatial scale also influences visual perception; narrower streets with high walls provide a sense of enclosure, whereas wider roads attract greater visual attention and facilitate better spatial recognition.

Therefore, in narrower streets, wall murals, wayfinding systems, or landscape walls can be utilized to enhance spatial recognition and visual attention. In wider streets, pedestrian promenades or separated shared pathways can be introduced to mitigate the sense of unease caused by excessive width. Additionally, increasing the height of roadside walls, buildings, or landscape greenery can further enhance people’s sense of security.

(2) Design elements and materials in relation to spatial perception and visual preferences

The experimental results indicate that: first, aesthetic and functional elements, such as vertical signage, landscape walls, rest areas, and abundant greenery, significantly improve the attractiveness, openness, and publicness of rural streets. Material combinations featuring vibrant colors (e.g., red and green) and natural textures (e.g., wood, stone, and relief bricks) enhance the perceived friendliness and safety of the space, encouraging people to linger and engage. Second, streets enriched with diverse public facilities and vibrant design elements draw more visual interest and engagement. Preferred material and color combinations, such as yellow relief bricks, wood, and greenery, consistently attract prolonged attention and positive evaluations. And finally, eye-tracking metrics, including gaze duration and saccade patterns, reveal strong correlations with subjective impressions of safety, comfort, and spatial appeal, validating the use of these methods to quantify emotional responses. Spaces that combine aesthetic appeal with safety are perceived as more public-friendly and inviting, ultimately increasing their ability to foster prolonged social and leisure activities.

Thus, in rural street design, vertical signage can be placed at intersections or key points to enhance spatial guidance; landscape walls can be set along the streets as boundaries between public and private spaces; and seating areas and flower beds can be arranged on both sides of the street to create focal points for visual attention, encouraging people to rest and stay. In terms of materials and colors, the use of wood, vibrant yet harmonious color combinations, and rich surface textures can enhance the sense of friendliness and security. Regarding spatial layout, designing areas conducive to gathering and interaction, such as circular seating or open rest areas, can promote social engagement. A flexible spatial layout can support various social and leisure activities, enhancing vibrancy and increasing the duration of stay. Additionally, integrating visually striking elements such as sculptures or murals can guide people’s gaze and movement, encouraging longer stays. Furthermore, utilizing eye-tracking technology to provide data support ensures the effectiveness of these design strategies.

In conclusion, the findings underscore the importance of carefully considering spatial scale, design elements, and material composition in rural street design. By aligning these factors with users’ emotional and behavioral needs, designers can create rural street spaces that are not only functional and aesthetically pleasing but also socially engaging and emotionally satisfying. This research provides valuable insights for enhancing the livability and vibrancy of rural environments through thoughtful design interventions. This study has several limitations. First, the sample size (n = 33) and its scope cannot fully encompass people from all regions, especially those with different cultural or geographical characteristics. Future research should include larger and more diverse samples to validate these findings. Second, the study focused on a single village in the North China Plain, which may limit the generalizability of the results. Additional studies in other rural contexts are needed to explore emotional differences across cultural and regional settings. Finally, the use of eye-tracking technology, while innovative, may not capture all aspects of emotional experience. Future research could combine eye-tracking with other methods, such as interviews or physiological measurements, to provide a more comprehensive understanding of emotional responses.

## Supporting information

S1 File Data. (XLS)
